# Behavioral and environmental drivers of dairy performance in alpine grazing cows managed with precision livestock farming technologies

**DOI:** 10.3168/jdsc.2025-0956

**Published:** 2026-03-12

**Authors:** Marta Pavolini, Giulia Gislon, Maddalena Zucali, Alberto Tamburini, Anna Sandrucci

**Affiliations:** Department of Agricultural and Environmental Sciences—Production, Landscape, Agroenergy, University of Milan, Milan, Italy, 20133

## Abstract

Dairy farming on mountain pastures is a traditional activity that is declining due to the numerous challenges posed by mountain environments. For 2 summer seasons (2024 and 2025), the feeding behaviors of a herd of Bruna breed cows (20 lactating cows in 2024, 8 primiparous and 12 multiparous; 25 in 2025, 8 primiparous and 17 multiparous) on the Italian Alps were investigated, together with milk production, milk composition, and pasture quality and availability. Cows grazed rotationally across plots differing in pastoral value (PV), botanical diversity, forage yield, and nutritional composition. Feeding behavior was monitored using RumiWatch halters (ITIN+HOCH GmbH, Switzerland), and milk yield and composition, BCS, and locomotion scores were assessed biweekly. This study evaluated how pasture characteristics, feeding behavior, and animal-based factors influence milk yield (MY) and milk composition. Multiparous cows exhibited more rumination time and less eating time than primiparous and secondiparous animals, suggesting more efficient grazing strategies. Rumination showed a positive association with MY, whereas longer eating time characterized low-producing cows. Moreover, BCS, parity, and rumination were significant predictors of MY and milk composition. Cows with lower BCS produced less milk, with reduced fat and protein. Pasture characteristics also affected dairy performance: greater milk production was associated with greater Shannon diversity and the presence of legume species, whereas PV and forage yield showed complex and sometimes inverse relationships with MY. Crude protein content of the forage had limited influence on milk solids yield. Multiple correspondence analysis confirmed that high-producing cows clustered with greater rumination, greater body condition, higher dairy efficiency (kg milk/kg DMI), and higher parity number; whereas low-producing cows were associated with low BCS, lower rumination, greater eating time, and lower parity number. Overall, the results highlight that, under alpine grazing conditions, milk production depends on the interaction between feeding behavior, animal status, and pasture features. Precision livestock farming technologies, which provide data on behavioral and productive responses of grazing cows and pastures, enable a better understanding of efficiency in mountain farming systems by enhancing grazing management and sustaining dairy productivity in pasture-based systems.

## Highlights

• Higher rumination and lower eating times indicate more efficient grazing cows.
• Better body condition and higher parity support higher milk production.
• Botanically diverse and legume-rich pastures enhance milk yield under alpine conditions.
• Precision livestock farming tools improve the monitoring and management of grazing dairy cows.

## Graphical Abstract

Dairy farming in alpine summer pastures faces environmental and management challenges. This study investigated how pasture characteristics, animal traits, and feeding behaviors influence milk yield and composition in Bruna cows across 2 grazing seasons. Multiparous cows ruminated more and ate less than primiparous cows, indicating greater grazing efficiency. Parity, body condition score (BCS), milk urea, and rumination behavior emerged as key predictors of milk production. Pasture features also contributed: higher milk yield was linked to greater botanical diversity and legume presence, whereas pastoral value and forage yield showed complex relationships with milk yield. Both crude protein (CP) and neutral detergent fiber (NDF) had limited effects on milk yield but influenced fat and protein. Multiple correspondence analysis confirmed that high-producing cows clustered with higher rumination, better BCS, and multiparous status. Precision livestock farming technologies improved the understanding of efficiency in alpine grazing systems.

**Figure:**
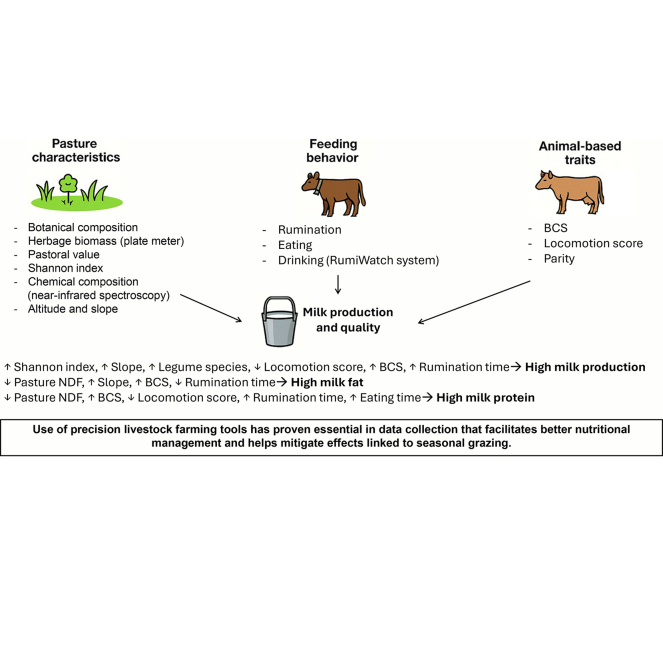

Dairy farming on mountain pastures during the summer season represents one of the few agricultural activities still practiced in the Italian Alps, despite the challenges that this harsh environment poses to animals and farmers. In recent decades, there has been a reduction in farming activities in the Alps, along with a decline in the practice of seasonal alpine grazing (Berton et al., 2021). Nevertheless, alpine grazing systems are important for preserving social and cultural traditions, for providing valuable products, and for maintaining ecosystem services (Mack et al., 2023). Moving dairy cows to mountain pastures, however, may imply disadvantages related to imbalanced diets, predation risks, and potentially reduced animal welfare (Franchini et al., 2025). High-altitude pastures may be insufficient to meet the nutritional requirements of the animals, with consequent negative effects. The ability to monitor feeding behaviors and pasture characteristics may be useful to understand animals' forage choices and intakes (Edwards et al., 2024). Precision livestock farming (**PLF**) tools enable the collection of extensive data, which can be used to improve nutritional management and minimize the negative effects associated with changing conditions (Marchegiani et al., 2025). The aim of the present study was to assess the effects of pasture availability and composition, animal-based traits, and feeding behaviors on milk yield (**MY**) and composition, through the use of PLF technologies, also evaluating how feeding behaviors are influenced by animal characteristics, such as parity number. The study was conducted over 2 summer seasons, 2024 and 2025, during which the same herd of Bruna cows was moved from the lowland to the mountain pastures (1,826 m above sea level), in Campodolcino Municipality, Sondrio, Italy (46°25′19.0″N, 9°21′36.1″E). The 2024 season lasted 68 d (Jun. 27–Sep. 3); and the 2025 season was 64 d (Jun. 25–Aug. 28). The total area grazed was 32.2 ha, classified in the Regional Land-Use Database (Dusaf 7.0, https://tinyurl.com/bdcaaws9) as “permanent pastures without trees and shrub species.” The herd consisted of 20 lactating cows in 2024 (8 primiparous, 12 multiparous) and 25 lactating cows in 2025 (8 primiparous, 17 multiparous). During the summer season, the animals were managed following a rotational grazing system, with new fenced grazing plots provided every evening after milking, according to grass availability. Cows were milked twice daily, using a mobile milking wagon. During each milking, all cows received 1.75 kg of supplementary feed (composition: 15.5% protein, 4% fat, 4.5% fiber, and 7% ash). Each year, 10 animals were equipped with a halter for monitoring feeding behaviors (RumiWatch, ITIN+HOCH GmbH, Switzerland): 5 and 4 primiparous cows in 2024 and 2025, respectively, and 5 and 6 multiparous. The RumiWatch halters were mounted on the animals' heads: data were recorded continuously over a 68- or 64-d period (2024 and 2025, respectively), with a scheduled dismounting at d 30 for data download and battery replacement, followed by immediate remounting of the devices. The behaviors recorded from the devices were processed using the RumiWatch Converter V7.3.36 and summarized as daily percentage of ruminating time (**rum%**), eating (both head up and head down; **eat%**), drinking (**dri%**), and other activity time. Milk data were collected every 2 wk, for a total of 10 milk collections (5 in 2024 and 5 in 2025). Individual MY was assessed during 2 consecutive milkings (evening, morning), and milk samples were then analyzed for fat content (kg; **MF**), protein content (kg; **MPro**), SCC (cells/mL) and urea (mg/dL; MUN). Moreover, every 2 wk, the cows were evaluated for BCS, following Ferguson et al. (1994), and for presence of lameness through locomotion scoring (**LS**; Sprecher et al., 1997), both assessed by trained operators. During the biweekly visits to the alpine farm, visual assessments of the botanical composition of the pastures were performed, quantifying the percentage contribution of each plant species to the sward (Braun-Blanquet, 1932). The sampling plots were selected according to the rotational schedule of the pasture: the next plots (3 to 4) that the farmer indicated would be opened for grazing were evaluated. Herbage biomass was estimated using a rising plate meter (**RPM**; GrassHopper, True North Technologies), to quantify the available herbage mass before grazing. A species-specific index (Roggero et al., 2002) was used to evaluate the pastoral value (**PV**) of each pasture plot. Species richness (**SR**; number of species) and Shannon index (**SH**; Shannon, 1948) were calculated to analyze the biodiversity of plant communities for each plot. Fresh grass samples were also collected and later analyzed using near-infrared spectroscopy (AuroraNIR, GraiNit, Italy) to determine chemical composition (DM, CP, NDF, NFC). For each plot, coordinates were derived using GPS Status & Toolbox app (v. 11.4.316; MobiWIA Kft.), and topographic features (altitude and slope) were then obtained using ArcGIS Pro 3.5 (ESRI) and the Lombardy regional digital terrain model (DTM 5X5 - Modello digitale del terreno ed. 2015; https://tinyurl.com/35f59v6n). Statistical analysis was performed using SAS 9.4 (SAS Institute Inc.), including descriptive statistics and a general linear model (**GLM**) for assessing the effect of number of parities on daily feeding behaviors:

[1] Y = μ + parity + ε,

where Y = response variable (rum% and eat%); µ = overall mean; parity = number of lactation classes (primiparous = 1, secondiparous = 2, multiparous >2); and ε = residual error.

Another GLM was used for evaluating the effects on fat- and protein-corrected milk (**FPCM**; IDF, 2015), MF, and MPro. The variables were divided into classes based on their median values (**Mdn**). The model used was as follows:

[2] Y = μ + PV + SH + CP + NFC + DM_Pasture_ + leg + slo + BCS + LS + MUN + rum% + eat% + dri% + parity + ε,

where Y = response variable (FPCM, MF, MPro); µ = overall mean; PV = pastoral value class (Mdn = 26.6); SH = Shannon class (Mdn = 1.96); CP = CP class (Mdn = 13.27); NFC = NFC class (Mdn = 26.1); DM_Pasture_ = DM yield, kg/ha of pasture class (Mdn = 3,183); leg = percentage of leguminous species class (Mdn = 3.92); slo = slope class (Mdn = 36.2); BCS = BCS class (BCS_Low <2.5, BCS_Medium 2.5–3, BCS_High >3); LS = locomotion class (LS_NonLame ≤2, LS_Lame >2); MUN = milk urea class (Mdn = 20.9); rum% = percentage of daily rumination class (Mdn = 0.26); eat% = percentage of daily eating class (Mdn = 0.44); dri% = percentage of daily drinking class (Mdn = 0.01); parity = number of lactation classes (primiparous = 1, secondiparous = 2, multiparous >2); and ε = residual error.

Multiple correspondence analysis (**MCA**) was carried out using RStudio (ver. 2024.12.0; R Foundation for Statistical Computing) to determine the relationships between milk production levels (low MY ≤15.6, 15.6 < medium MY ≤ 19.5, high MY >19.5 kg/d) and animal-based variables (parity, BCS, dairy efficiency [**DE**]), pasture variables (PV, DM yield, kg/ha of pasture [**DM_Pasture_**]), and activity variables (rum%, eat%). The MCA (FactoMineR package) was used to identify associations between MY classes and pasture, behavioral, and animal-related variables, grouping observations with similar relationships into color-coded classes. For calculation of DE, pasture DMI was computed according to Vazquez and Smith (2000). Differences were considered significant at *P* ≤ 0.05.

Average daily MY was 16.9 kg (SD 4.37), with 4.53% (SD 0.60%) fat and 3.54% (SD 0.37%) protein. Average BCS was 2.71 (SD 0.59), similar to the findings of Zendri et al. (2016). Pastures ranged from 1,767 to 1,936 m above sea level, with an average 4% of tree cover (SD 6.82%) and 10.6% presence of shrubs (SD 11.35%), and a predominance of botanical species with null PV (*Chaerophyllum*, *Nardus*, and *Rumex*). The average PV across all evaluated plots was 28.6 (SD 10.6, from 8.95 to 50.2), showing a decrease with increasing elevation, as observed by Pittarello et al. (2018) for the Piedmont region. Similarly, SR was in line with their findings for alpine pastures (29.9 species, SD 7.35). In contrast, SH indicated lower biodiversity compared with Pittarello et al. (2018), but it was consistent with the results of Cislaghi et al. (2019) for a valley close to the one examined in this study. As observed by Cislaghi et al. (2019), the dominance of *Nardus* may contribute to a reduction in the biodiversity of the plots. Nutritional composition results for DM_Pasture_ and CP are in line with the results of Cislaghi et al. (2019), whereas NDF was largely lower (48.1%, SD 3.18%). It should be noted that the composition of mountain pastures reported in the literature is quite variable: several authors found higher NDF content than the results obtained in the present study (Leiber et al., 2006; Koczura et al., 2019). It should also be highlighted that the near-infrared analysis for alpine mixed pasture species may not be fully accurate for all components, and that local calibration is suggested (Confalonieri et al., 2004; Boschetti et al., 2007). Pasture biomass was assessed with an RPM due to its ease of use, and the average DM_Pasture_ was 2.8 t DM/ha (SD 1.28), similar to the results of Cavallero et al. (1992) for the same alpine region. Calibration of the RPM model for estimating biomass in specific grassland types, especially those located on steep slopes such as those under examination, still needs further investigation (Mack et al., 2023). The average time cows spent ruminating was 6.2 h/d (SD 1.7 h), shorter than the values observed for animals kept indoors (Hut et al., 2022). Instead, the animals on pasture showed, as expected, a longer time spent on eating behavior, for a total of 42.8% (SD 6.88%) of the day spent grazing and chewing, corresponding to ~10.3 h/d spent on eating activity, similar to the observations of Rombach et al. (2019).

Evaluation of the effects of parity on feeding behavior (obtained from Eq. 1) showed that rumination duration was higher in multiparous cows (*P* < 0.01) compared with those in their first and second lactations (Figure 1B). This result contrasts with the findings of Macmillan et al. (2022), who did not report differences in rumination time associated with parity. Conversely, primiparous and secondiparous cows spent more time eating during the 2 seasons examined, compared with multiparous ones (*P* < 0.01; Figure 1A). The shorter time spent eating by multiparous cows could be due to a different feeding strategy, as observed indoors by Beauchemin (2018), where multiparous cows tended to eat more in each meal compared with animals in their first lactations. Florit et al. (2023) also found that, from the third lactation onward, cows showed lower eating time, whereas younger animals ate a larger number of separate meals for a greater total time spent eating (Hut et al., 2022), because of smaller meal size per visit at the manger. Similarly, Azizi et al. (2010) reported reduction in eating duration with increasing number of parities. Multiparous animals appear to make more efficient use of pastures, spending less time eating, and ruminating more. Increased rum% combined with reduced eat% may indicate that they achieve adequate energy intake through fewer, larger meals and more complete fiber breakdown during rumination.

**Figure 1:**
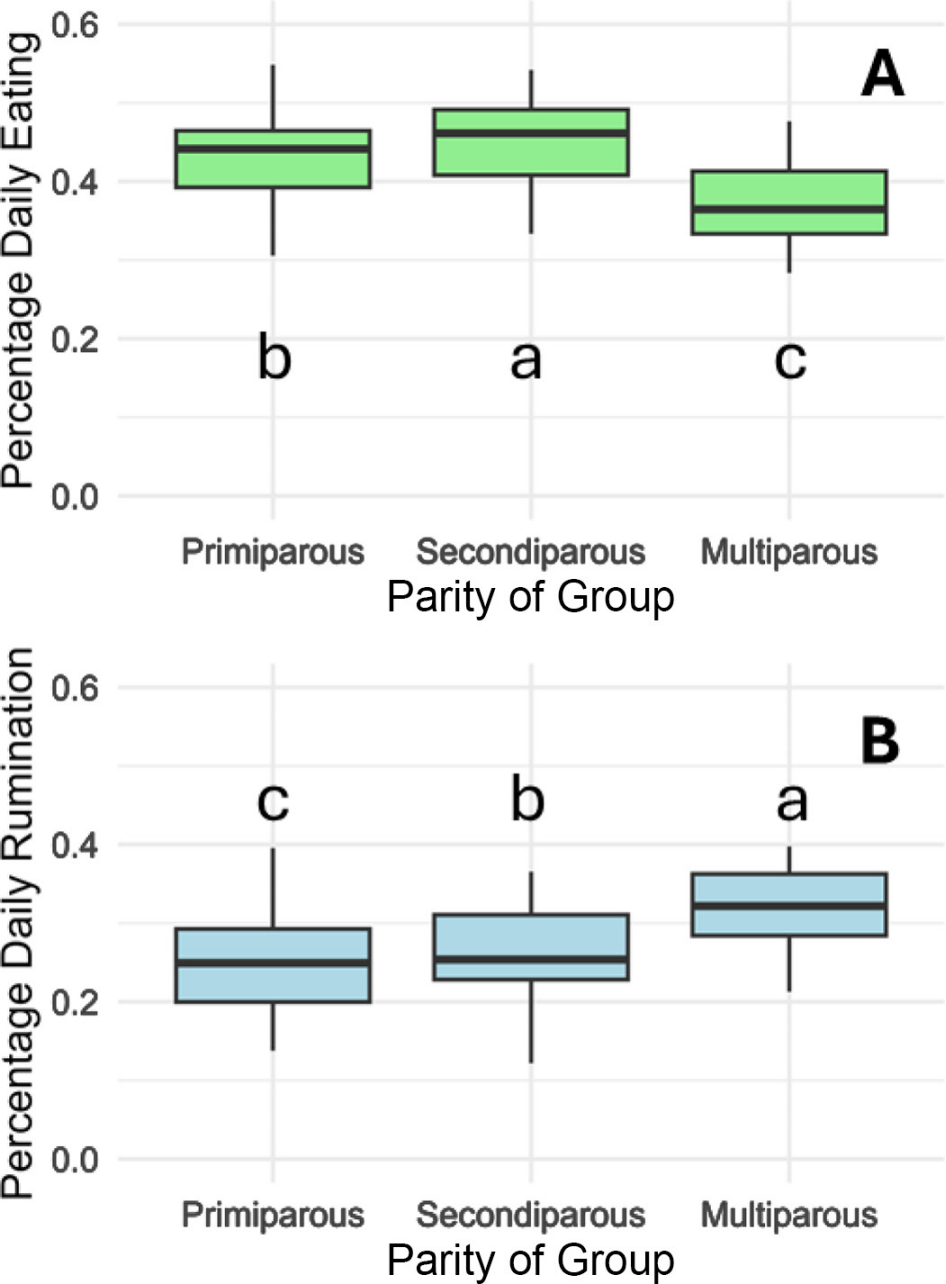
Effects of parity on daily eating (A) and rumination (B) percentages in Italian alpine grazing cows. Upper and lower edges represent the 25th to 75th percentile, and bold midlines represent the median values. Lowercase letters (a–c) indicate differences among parity groups (A: *P* < 0.01; B: *P* < 0.03).

The most relevant results of the GLM (Eq. 2), performed to assess the influence of multiple parameters on MY, MF, and MPro, are shown in Table 1. The present study highlighted that daily MY was influenced by PV, SH, NFC, DM_Pasture_, and presence of legume species (*P* = 0.03, *P* < 0.01, *P* = 0.03, *P* < 0.01, *P* < 0.01, respectively). It was also affected by slope, unexpectedly showing higher MY (*P* < 0.01), MF (*P* < 0.01), and MPro (*P* < 0.01) from plots with higher degrees of inclination. It is possible that steeper slopes experience less trampling pressure from the grazing animals, remaining richer in plant species and more fertile for forage growth (Probo et al., 2025) and, therefore, more valuable for milk production. Pastures associated with low PV resulted in higher MY, expressed as FPCM. Greater SH was associated with higher MY, confirming that greater botanical biodiversity has positive effects on dairy performance (Roca-Fernández et al., 2016). The amount of pasture CP did not seem to affect any of the response variables (FPCM, MF, and MPro; *P* = 0.29, 0.21, and 0.56, respectively). Instead, NFC content influenced FPCM and MPro (*P* = 0.03, *P* < 0.01). Lower concentrations of NFC in the forages were associated with higher MY and MPro, whereas studies conducted on confined animals reported no influence or slight increase in FPCM, MF, and MPro with diets richer in carbohydrates (i.e., NFC; Martins et al., 2021). However, lower values of NFC are usually associated with higher amounts of structural components (NDF) in the diet, which can increase rumination duration, and in turn, may enhance MY and MPro (Hristov and Ropp, 2003; Souza et al., 2022), as observed in the present study. The measured DM_Pasture_ indicated higher MY from pastures with lower DM_Pasture_ (18.9 vs. 17.5 kg FPCM/d, *P* < 0.01). It should be noted that pasture samples, which were cut at 4 cm from the ground, likely included some senescent material from the previous season. This residual part could contribute to higher DM, but with lower nutritive value. The consequence may be a reduction in the digestibility of the forages potentially increasing the filling of the rumen, thus also negatively influencing MY (Leiber et al., 2006). Legume species have a well-known positive effect on MY (*P* < 0.01; Johansen et al., 2018), confirming that the presence of the family *Fabaceae* could contribute to better dairy performance of grazing cows. Regarding the animal-based variables, LS and BCS affected animals' production: lame cows showed a tendency for lower MY compared with non-lame ones (*P* = 0.02). Meanwhile, BCS showed strong effects on MY (*P* < 0.01) and MF and MPro (*P* < 0.01). Cows with lower BCS produced less milk with lower MF and MPro. It is plausible that grazing animals with lower body condition mobilized their body reserves for sustaining milk production, resulting in a decrease in BCS, which was not compensated for by the pasture-based diet they rely on (Roche et al., 2007). The content of urea in milk was also a driver of all response variables, with greater amounts of MUN being associated with higher MY, MF, and MPro. This result may reflect the fact that animals with higher MY generally have greater nutrient requirements and feed intake, which increase nitrogen intake that may be subsequently released in milk (Zhao et al., 2025). Daily rumination (rum%) affected MY (*P* < 0.01), and a possible correlation between rumination time and milk production has been suggested by Byskov et al. (2017). A positive relation between rumination time and MY was observed, with longer rumination duration resulting in highest milk production, confirming the association already found by Stone et al. (2017). Low rumination time was commonly associated with a decrease in MF, in accordance with the observations of the present study, where a decrease in MF was observed when the cows ruminated for shorter time (*P* < 0.01). In contrast, no relation was found between MF and rumination time by Andreen et al. (2021), whereas some weak influence was reported in the study of Johnston and DeVries (2018). Moreover, the present study found no association between eating time and MY, despite effects of eating time on MY found under barn conditions by other authors (Johnston and DeVries, 2018; Codl et al., 2023). Some authors found a negative relation between eat% and MPro, although in the present study the data collected on eating time did not suggest any strong relation with MY, MF, or MPro (*P* = 0.59, 0.27, and 0.45, respectively). Also, parity was found to affect MY (*P* < 0.01), MF (*P* < 0.01), and MPro (*P* < 0.01), with greater MY in cows with more than 1 lactation, and higher MF and MPro in those in their second lactation. Similar results were also found by Codl et al. (2023), who observed an increase in milk production in cows with increasing numbers of lactations.

**Table 1:** Influence of pasture quality factors, pasture availability, nutritional parameters, pasture topography, animal welfare indicators, behavioral factors and parity number on daily milk yield (as FPCM; kg), fat content (kg), and protein (kg) content (LSM; FPCM SE: 0.62; MF SE:0.03; MPro SE: 0.02)

Variable	Class	Median value (Mdn)	Unit of measure	Individual daily milk production (FPCM), kg	Milk fat (MF), kg	Milk protein (MPro), kg	*P*-value FPCM	*P*-value MF	*P*-value MPro
Pastoral value (PV)	PV_Low	≤26.6	—	18.59	0.79	0.59	0.03	0.08	<0.01
	PV_High	>26.6	—	17.80	0.76	0.56			
Shannon index (SH)	SH_Low	≤1.96	—	17.11	0.73	0.54	<0.01	<0.01	<0.01
	SH_High	>1.96	—	19.28	0.82	0.61			
Pasture CP	CP_Low	≤13.27	% DM	18.37	0.78	0.58	0.29	0.21	0.56
	CP_High	>13.27	% DM	18.02	0.76	0.57			
Pasture NFC	NFC_Low	≤26.1	% DM	18.56	0.78	0.59	0.03	0.25	<0.01
	NFC_High	>26.1	% DM	17.83	0.76	0.56			
Pasture yield (DM_Pasture_)	DM_Pasture__Low	≤3.18	t DM/ha	18.91	0.81	0.60	<0.01	<0.01	<0.01
	DM_Pasture__High	>3.18	t DM/ha	17.48	0.74	0.56			
Legume presence (leg)	leg_Low	≤3.92	%	17.68	0.75	0.56	<0.01	<0.01	<0.01
	leg_High	>3.92	%	18.71	0.79	0.59			
Slope (slo)	slo_Low	≤36.2	%	17.08	0.72	0.55	<0.01	<0.01	<0.01
	slo_High	>36.2	%	19.31	0.83	0.61			
BCS	BCS_Low	<2.5	—	17.13	0.73	0.53	<0.01	<0.01	<0.01
	BCS_Med	2.5–3	—	18.66	0.78	0.59			
	BCS_High	>3	—	18.80	0.81	0.61			
Locomotion score (LS)	LS_NonLame	≤2	—	19.05	0.80	0.63	0.02	0.16	<0.01
	LS_Lame	>2	—	17.34	0.75	0.53			
Milk urea (MUN)	MUN_Low	≤20.9	mg/dL	17.24	0.72	0.56	<0.01	<0.01	<0.01
	MUN_High	>20.9	mg/dL	19.15	0.83	0.59			
Daily rumination time (rum%)	rum%_Low	≤0.26	%	16.68	0.71	0.54	<0.01	<0.01	<0.01
	rum%_High	>0.26	%	19.71	0.84	0.61			
Daily eating time (eat%)	eat%_Low	≤0.44	%	18.28	0.78	0.58	0.59	0.27	0.45
	eat%_High	>0.44	%	18.11	0.76	0.57			
Parity	Primiparous	1	—	15.31	0.65	0.48	<0.01	<0.01	<0.01
	Secondiparous	2	—	20.31	0.87	0.64			
	Multiparous	>2	—	18.96	0.80	0.61			

Results obtained from MCA are shown in Figure 2. The first 2 dimensions of the MCA explained a total of 37.2% of the variance. The plot showed that high-producing cows (HiMY) clustered mainly in the area associated with higher rumination (rum%_High) and lower feeding time (eat%_Low), higher BCS (BCS_high), higher dairy efficiency (DE_High; DE >1.10), and multiparous status. These associations indicate that high MY was more linked with higher body condition and more efficient feeding behavior, rather than with favorable environmental conditions (PV_High and DM_Pasture__High). Conversely, low-producing cows (LoMY) were associated with lower BCS (BCS_Low), lower rumination (rum%_Low), higher eating time (eat%_High), lower dairy efficiency (DE_Low; DE ≤1.10), and younger animals (first or second lactation), suggesting less efficient feed utilization. Overall, the MCA supports the results of the GLM analyses by confirming that both behavioral (rumination, eating) and environmental variables (PV, DM_Pasture_) jointly contribute to determining MY. The clear gradient observed across the FPCM classes underscores the role of feeding behaviors and parity as key determinants of dairy performance.

**Figure 2:**
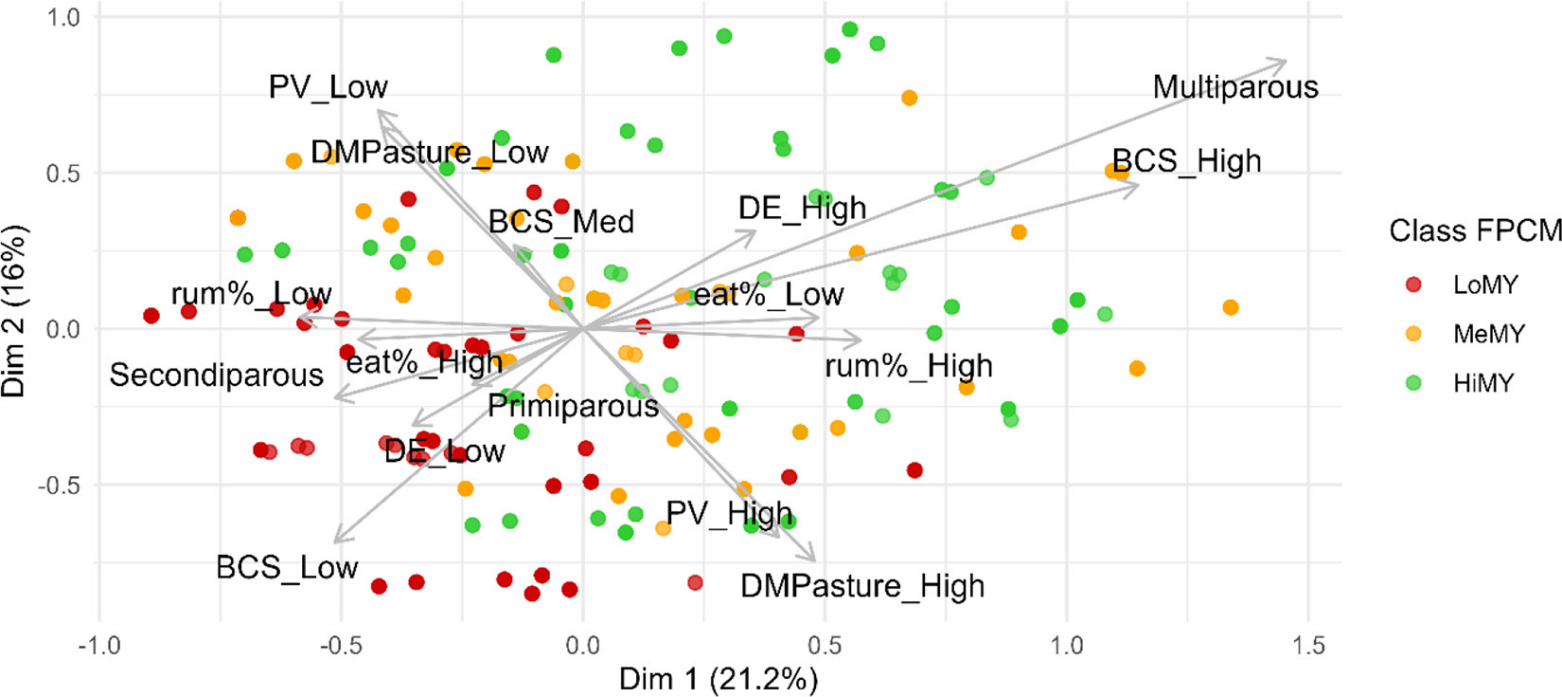
Multiple correspondence analysis (MCA) showing associations among pasture, behavioral, and productive variables. Dim = dimension; LoMY ≤15.6, 15.6 < MeMY ≤ 19.5, HiMY >19.5 kg/d = high, medium, and low milk yield classes, respectively; see Table 1 for other variable classes.

In conclusion, this study showed that milk yield and composition of Bruna cows on alpine pastures are jointly influenced by feeding behavior, animal traits, and pasture characteristics. Multiparous cows and those with higher rumination times produced more milk, whereas low-producing cows showed lower BCS, reduced rumination, and longer eating duration. Botanical diversity and the presence of legume species were positively associated with milk production, although deeper investigation is needed for a better understanding of the influence of forage nutritional traits. Use of PLF tools has proven essential in data collection that facilitates better nutritional management and helps mitigate the effects linked to seasonal grazing. Further research aimed at improving pasture management and milk productivity will be necessary for ensuring the viability of dairy farming in alpine systems.
